# Knowledge and Attitude of Healthcare Providers Regarding Palliative Care and Related Factors: An Online Cross-Sectional Study

**DOI:** 10.7759/cureus.54477

**Published:** 2024-02-19

**Authors:** Sarya Swed, Haidara Bohsas, Hidar Alibrahim, Khaled Albakri, Mohammed Amir Rais, Mohammad Badr Almoshantaf, Wael Hafez, Yasmeen Abouainain, Bisher Sawaf, Lamees Alshareef, Zain Alabdeen Ayman Othman, Ibrahim Elbialy, Hekmieh Manad, Youmna Faheem, Steffi John, Jalal Alshareef, Lana Sheet, Amine Rakab

**Affiliations:** 1 Medicine, University of Aleppo, Aleppo, SYR; 2 Internal Medicine, University of Aleppo, Aleppo, SYR; 3 Faculty of Medicine, Hashemite University, Zarqa, JOR; 4 Dentistry, University of Algiers, Algiers, DZA; 5 General Surgery, Ealing Hospital, London, GBR; 6 Internal Medicine, NMC Royal Hospital, Abu Dhabi, ARE; 7 Internal Medicine, National Research Centre, Cairo, EGY; 8 Medicine, University of Jordan, Faculty of Medicine, Amman, JOR; 9 Internal Medicine, Syrian Private University, Faculty of Medicine, Damascus, SYR; 10 Faculty of Medicine, University of Aleppo, Aleppo, SYR; 11 Medicine, Al-Baath University, Faculty of Dentistry, Homs, SYR; 12 Internal Medicine, Burjeel Hospital, Abu Dhabi, ARE; 13 Internal Medicine, Mediclinic Hospital, Abu Dhabi, ARE; 14 College of Medicine, Ras Al Khaimah Medical & Health Sciences University, Ras Al-Khaimah, ARE; 15 College of Medicine, NMC Royal Hospital, Abu Dhabi, ARE; 16 Pediatrics, California Institute of Behavioral Neurosciences and Psychology, Fairfield, USA; 17 Obstetrics and Gynaecology, NMC Royal Hospital, Abu Dhabi, ARE; 18 Medical Education, Weill Cornell Medicine – Qatar, Education City, QAT

**Keywords:** cross-sectional study, healthcare workers, attitudes, knowledge, palliative care

## Abstract

Background: Palliative care alleviates pain and enhances the quality of life of patients with life-threatening illnesses. Training programs are required to provide patients with proper care and advance their health because the expertise of healthcare personnel in palliative care is inadequate.

Aim: We aimed to assess healthcare professionals' knowledge of palliative care because palliative care programs are infrequently used in Syria.

Methods: An online cross-sectional study was conducted between July 24, 2022, and August 28, 2022, to assess palliative care knowledge and applications among Syrian healthcare workers. The study questionnaire was designed in accordance with a previous study, and the inclusion criteria included Syrian healthcare workers, physicians, and nurses, as well as medical and nursing students. The first section of the questionnaire included sociodemographic information, while the second, third, and fourth sections assessed healthcare workers' experiences, knowledge, and attitudes toward palliative care, respectively.

Results: Of the 602 participants, 66.2% of the sample study were females. The majority of the respondents (72.9%) were medical students, with 18.8% residents and 8.3% nurses or nursing students. The majority of the participants (84%) correctly answered the question about pain treatment goals, while only a small percentage (5.3%) correctly answered the question about whether long-term opioid use was addictive. There were no statistically significant differences in the overall knowledge levels across demographic areas, genders, or specialties. Only 14 participants were considered knowledgeable about palliative care. Regarding attitudes toward palliative care, the three responses that received the greatest degree of agreement were "Pain relievers should be given as needed to terminally ill patients" (89.7%) and "Patients have the right to determine their own degree of psychosocial intervention" (81%). Residents in urban and rural areas scored markedly different in their attitudes. Students in their fifth year were 8.06 times more likely to have a positive attitude when compared to those in their first year.

Conclusions: Our findings show that Syrian healthcare providers lack knowledge of palliative care. It is important to integrate palliative care into Syria's healthcare system to enhance the quality of life of patients who are approaching the end of their lives and to provide care for those who require it.

## Introduction

Palliative care is an intervention that reduces pain and improves the quality of life for those coping with the consequences of life-threatening diseases [1]. It was only 40 years ago that palliative care played a central role in oncology medicine as an instrument for relieving pain and reducing the severity of symptoms related to advanced malignant illness [2]. According to studies, 40 million people worldwide need palliative care as a part of their treatment regimen; around 34% of these patients have cancer, and the majority live in low-and middle-income countries. It is thought that palliative care is offered to only 14% of patients with cancer who need it [3].

Globally, the need for palliative care has increased due to various factors, including the growing incidence of multimorbidity, the increasing number of patients living independently, and the broad acknowledgment of early referral to palliative care. By 2060, it is estimated that 48 million individuals will die annually owing to severe health conditions [4]. Palliative care aims to reduce pain and other symptoms, make patients as comfortable as possible in their last days, satisfy the needs of patients and their families via the collaborative efforts of a multidisciplinary team, and provide bereavement assistance when necessary.

Palliative care is beneficial in the early stages of diseases; it may also be used with curative therapies, such as chemotherapy and radiation, to improve the survival rates of end-stage diseases [5]. Palliative care providers included physicians, nurses, social workers, pharmacists, physiotherapists, and occupational therapists. They can work together in various settings, from hospitals and clinics to nursing homes and patients' homes [6]. Most intensive care unit (ICU) patients experience intense, unrelenting pain. Proactive palliative care in the intensive care unit and other counseling or integrative therapies may shorten hospitalization time and length of stay in the intensive care unit but do not affect mortality [7].

Furthermore, palliative care has shown significant benefits in the ICU because it incorporates the treatment of various symptoms in critically ill patients, including physical, psychological, and spiritual disorders, as well as communication regarding care goals, clinician and family support, and planning for care transition [8]. It has been demonstrated that palliative care benefits non-cancer patients with chronic diseases, such as heart failure, chronic obstructive pulmonary disease (COPD), and dementia, and decreases the quality of life by reducing the need for emergency room visits and the severity of their symptoms. However, it did not improve the quality of life [9]. The barriers to palliative care include moral tensions between what healthcare practitioners can do and what they want to offer. Physicians’ attitudes about palliative care, communication difficulties, and poor collaboration among healthcare professionals impede palliative care [10].

Observational studies have shown that the public's knowledge of palliative care is insufficient. This gap has resulted in a decline in the demand for palliative care from patients with severe conditions and incorrect perceptions of palliative care, leading patients to have less confidence in the application of palliative care [11,12]. Studies show a lack of knowledge regarding palliative care among medical professionals, which suggests that training programs for these workers should be improved to provide patients with appropriate medical treatment [13,14].

In Syria, where palliative care programs are rarely applied, it is essential to take a close look at the awareness of this subject. Accordingly, this study aimed to assess Syrian healthcare workers' knowledge of palliative care to improve the health of patients with life-threatening conditions.

## Materials and methods

An online cross-sectional study was conducted in Syria from July 24 to August 28, 2022, to assess Syrian healthcare professionals' knowledge of palliative care and its applications. Healthcare workers from all Syrian provinces, including physicians, nurses, medical students, and nursing students, were eligible. Participants from outside the medical field and those of non-Syrian nationalities were excluded from this study. All participants were informed of the purpose of the study, the identity of the teamwork, their right to withdraw from the study, the complete confidentiality of their personal information, and the fact that only data that were completely reported would be examined.

This study was created using a comprehensive questionnaire verified based on prior research [15]. The questionnaire was then translated into Arabic for the respondents’ understanding. A Google form questionnaire was created out of concern for security and distributed to the respondents via Facebook, WhatsApp, and Telegram. The government’s public places, such as hospitals, shopping centers, parks, public squares, and other public gathering places, as well as face-to-face interviews, were used to collect data.

Using a single proportion of the population formula, the minimum sample size was determined [n = [(Zα/2)2. P (1-P)]/d2], We used a population proportion (P) of 50%, a margin of error of 5%, a 95% confidence level (Z/2 = 1.96), and an additional 5% for the non-response rate. The final sample size was set to 385.

Measures

The items in this questionnaire were based on previous reviews and expert discussions, and they were used as an assessment tool to assess healthcare providers' knowledge of palliative care [16-19]. The questions were then divided into four groups. The first section contained information on the sociodemographic variables of the participants. The second section assessed healthcare providers' experience with patient care. The third domain is divided into five subsections and includes an examination of healthcare professionals' knowledge of palliative care (philosophy, pain, dyspnea, psychiatric problems, gastrointestinal problems, and communication). The fourth section asked the participants about their attitudes toward palliative care. A scale of 0-20 was used to assess healthcare professionals' knowledge of palliative care. These points were used to calculate the final score. Each correct answer is worth one point, and each incorrect answer is worth zero points.

Sociodemographic Variables

This section consisted of 11 questions, including age, marital status, and educational status, as the participant's primary demographic data (whether the respondent was a physician, nurse, or medical student). The participants were also asked how long they had been practicing as clinicians (less than or more than five years). Medical students were asked about their year of study, while doctors and nurses were asked about the hospital department in which they worked.

Experience of Healthcare Providers Regarding Palliative Care

The five questions in this section asked about the number of cancer patients that the participants have ever cared for, their experience working in a progressive care unit, how much time they spend learning about palliative care, and whether they have a mentor who can offer advice on end-of-life matters.

Knowledge of Caregivers Regarding Palliative Care

This section consisted of 20 questions designed to assess general palliative care knowledge. This section is divided into five subdomains: philosophy, pain, dyspnea, psychiatric problems, gastrointestinal problems, and palliative care communication. The ideas discussed in this section include whether palliative care is only for non-curative diseases, whether getting a good night's sleep is one of the primary goals of palliative care, and whether dyspnea caused by cancer can be treated with morphine or other opioids. The respondents were also asked about their thoughts on the use of benzodiazepines to treat delirium and whether steroids could increase appetite in patients with cancer.

Attitude of Caregivers Regarding Palliative Care

Fifteen evaluation questions were provided in this section to evaluate the attitudes of healthcare professionals toward palliative care. In this section, the participants were asked whether they agree or disagree with the following statements: "end-of-life pain is a normal part of dying"; "palliative care should be the standard medical treatment for terminally ill patients"; "oral morphine addiction is a serious problem given that terminally ill patients have a short time to live"; and "the primary caregiver is the most appropriate person to make end-of-life decisions and take appropriate measures." The participants were asked whether they think that people who are terminally ill should be kept pain-free and whether it is best to avoid talking about death with people who are close to the end of their lives.

Pilot study

To show the validity and comprehensibility of the questionnaire, we sent it to 50 randomly chosen members of the general public. After conducting a pilot study and confirming the high levels of internal consistency (Cronbach's alpha ranged from 0.712 to 0.861), we distributed the online survey to start data collection.

Ethical considerations

The Syrian Ethical Society for Scientific Research approved this project on ethical grounds. Aleppo University provided ethical clearance. The participants were given a URL to access the Google online survey and were asked on the first page if they agreed to complete the questionnaire. The participants were directed to a linked page containing important research information before completing the questionnaire. The survey lasted for five to 12 minutes.

Statistical analysis

For the statistical analysis, the IBM SPSS Statistics for Windows, version 28 (released 2021; IBM Corp., Armonk, New York, United States) package application was used. Statistical significance was set at p < 0.05. Frequencies and descriptive statistics were used to represent categorical variables describing the parents' socioeconomic status. We conducted a univariate analysis using the Mann-Whitney U-test (for non-normal continuous variables), t-test (for normally distributed continuous variables), and chi-squared test to examine the factors that influence the knowledge and attitude toward palliative care of Syrian medical students and healthcare providers (for categorical variables). The odds ratios of an adequate level of knowledge of palliative care were then evaluated using multivariate logistic regression analysis for the significant factors (p = 0.05) in the univariate study.

## Results

The study included 602 participants, with an average age of 23.7 years old. Around two-thirds of the participants (66.2%) were female, with the majority being single, living in cities, and having chronic diseases (89.7%, 71%, and 87.7%, respectively). Medical students were most likely to respond (72.9%), followed by resident doctors (18.8%) and nurses or nursing students (8.3%) (Table 1).

**Table 1:** Sociodemographic characteristics of the participants *This variable is represented by the mean (standard deviation (SD)).

Variables		Number (%) / Mean (SD)
Age*		23.7 (5.03)
City where you live	Damascus countryside	37 (6.2%)
	Suwayda	8 (1.3%)
	Aleppo	151 (25.1%)
	Damascus	115 (19.1%)
	Hama	75 (12.5%)
	Idlib	19 (3.2%)
	Raqqa	13 (2.2%)
	Homs	62 (10.3%)
	Daraa	70 (11.6%)
	Latakia	13 (2.2%)
	Tartous	16 (2.7%)
	Al-Hasakah	10 (1.7%)
	Der Alzoor	12 (2.0%)
Gender	Male	203 (33.8%)
	Female	398 (66.2%)
Social status	Single	539 (89.7%)
	Married	58 (9.7%)
	Divorced	3 (0.5%)
	Widowed	1 (0.2%)
Economic status	Bad	56 (9.3%)
	Average	212 (35.3%)
	Good	301 (50.1%)
	Excellent	32 (5.3%)
Living site	City	427 (71.0%)
	Rural area	174 (29.0%)
Having a chronic disease	Yes	74 (12.3%)
	No	527 (87.7%)
Specialties	Medical student	438 (72.9%)
	Resident	113 (18.8%)
	Nurse or nursing student	50 (8.3%)
Year of study (If you are a student)	First year	10 (2.3%)
	Second year	52 (12.0%)
	Third year	77 (17.8%)
	Fourth year	62 (14.4%)
	Fifth year	133 (30.8%)
	Sixth year	98 (22.7%)

The goal of pain management was the knowledge question with the highest percentage of correct answers (84.2%), while the knowledge question asking whether long-term opioid use would lead to addiction had the lowest percentage (5.3%). Males were less likely than females to be familiar with patients who should receive palliative care. Residents provided the most accurate response to the question about avoiding the combination of palliative care and anticancer therapies or nonsteroidal agents. Compared to other specialties, the question of whether morphine causes delirium in terminally ill cancer patients is widely known among nurses or nursing students. However, there was no detectable difference in the total education level between places of residence, sex, or specialty (Table 2).

**Table 2:** Factors affecting the knowledge-related answers of the participants *P value < 0.05

Questions	Correct answer	Gender		Specialty			Living site	
		Male	Female	Medical students	Residents	Nurse or nursing student	City	Rural area
Philosophy								
Palliative care should only be provided for patients who have no curative treatments available.	False: 332 (55.2%)	93.0 (45.8%)	239.0 (60.1%) *	240.0 (54.8%)	72.0 (63.7%)	20.0 (40.0%)	243.0 (56.9%)	89.0 (51.1%)
Palliative care should not be provided along with anti-cancer treatments.	False: 439 (73%)	143.0 (70.4%)	296.0 (74.4%)	321.0 (73.3%)	93.0 (82.3%)	25.0 (50.0%)*	323.0 (75.6%)	116.0 (66.7%)
Pain								
One of the goals of pain management is to get a good night’s sleep.	True: 506 (84.2%)	166.0 (81.8%)	340.0 (85.4%)	362.0 (82.6%)	104.0 (92.0%)	40.0 (80.0%)	372.0 (87.1%)	134.0 (77.0%) *
When cancer pain is mild, pentazocine should be used more often than an opioid.	False: 59 (9.8%)	21.0 (10.3%)	38.0 (9.5%)	43.0 (9.8%)	10.0 (8.8%)	6.0 (12.0%)	37.0 (8.7%)	22.0 (12.6%)
When opioids are taken on a regular basis, nonsteroidal anti-inflammatory drugs should not be used.	False: 80 (13.3%)	37.0 (18.2%)	43.0 (10.8%) *	49.0 (11.2%)	24.0 (21.2%)	7.0 (14.0%) *	57.0 (13.3%)	23.0 (13.2%)
The effect of opioids should decrease when pentazocine or buprenorphine hydrochloride is used together after opioids are used.	True: 115 (19.1%)	44.0 (21.7%)	71.0 (17.8%)	84.0 (19.2%)	22.0 (19.5%)	9.0 (18.0%)	79.0 (18.5%)	36.0 (20.7%)
Long-term use of opioids can often induce addiction.	False: 32 (5.3%)	16.0 (7.9%)	16.0 (4.0%) *	21.0 (4.8%)	7.0 (6.2%)	4.0 (8.0%) *	21.0 (4.9%)	11.0 (6.3%)
Use of opioids does not influence survival time.	True: 218 (36.3%)	64.0 (31.5%)	154.0 (38.7%)	153.0 (34.9%)	50.0 (44.2%)	15.0 (30.0%)	154.0 (36.1%)	64.0 (36.8%)
Dyspnea								
Morphine should be used to relieve dyspnea in cancer patients.	True: 100 (16.6%)	43.0 (21.2%)	57.0 (14.3%) *	69.0 (15.8%)	18.0 (15.9%)	13.0 (26.0%) *	66.0 (15.5%)	34.0 (19.5%)
When opioids are taken on a regular basis, respiratory depression will be common.	False: 68 (11.3%)	26.0 (12.8%)	42.0 (10.6%)	45.0 (10.3%)	14.0 (12.4%)	9.0 (18.0%)	46.0 (10.8%)	22.0 (12.6%)
Oxygen saturation levels are correlated with dyspnea.	False: 75 (12.5%)	25.0 (12.3%)	50.0 (12.6%)	50.0 (11.4%)	17.0 (15.0%)	8.0 (16.0%) *	49.0 (11.5%)	26.0 (14.9%)
Anticholinergic drugs or scopolamine hydrobromide are effective for alleviating bronchial secretions of dying patients.	True: 229 (38.1%)	80.0 (39.4%)	149.0 (37.4%)	169.0 (38.6%)	42.0 (37.2%)	18.0 (36.0%)	164.0 (38.4%)	65.0 (37.4%)
Psychiatric problems								
During the last days of life, drowsiness associated with electrolyte imbalance should decrease patient discomfort.	True: 182 (30.3%)	71.0 (35.0%)	111.0 (27.9%)	134.0 (30.6%)	27.0 (23.9%)	21.0 (42.0%)	120.0 (28.1%)	62.0 (35.6%)
Benzodiazepines should be effective for controlling delirium.	True: 261 (43.4%)	72.0 (35.5%)	189.0 (47.5%) *	194.0 (44.3%)	52.0 (46.0%)	15.0 (30.0%) *	188.0 (44.0%)	73.0 (42.0%)
Some dying patients will require continuous sedation to alleviate suffering.	True: 431 (71.7%)	135.0 (66.5%)	296.0 (74.4%)	297.0 (67.8%)	99.0 (87.6%)	35.0 (70.0%) *	315.0 (73.8%)	116.0 (66.7%)
Morphine is often a cause of delirium in terminally ill cancer patients.	False: 61 (10.1%)	24.0 (11.8%)	37.0 (9.3%)	32.0 (7.3%)	17.0 (15.0%)	12.0 (24.0%) *	42.0 (9.8%)	19.0 (10.9%)
Gastrointestinal problems								
At terminal stages of cancer, higher calorie intake is needed compared to early stages.	False: 129 (21.5%)	40.0 (19.7%)	89.0 (22.4%)	97.0 (22.1%)	25.0 (22.1%)	7.0 (14.0%)	87.0 (20.4%)	42.0 (24.1%)
There is no route except central venous for patients unable to maintain a peripheral intravenous route.	False: 63 (10.5%)	21.0 (10.3%)	42.0 (10.6%) *	43.0 (9.8%)	18.0 (15.9%)	2.0 (4.0%) *	45.0 (10.5%)	18.0 (10.3%)
Steroids should improve appetite among patients with advanced cancer.	True: 150 (25%)	52.0 (25.6%)	98.0 (24.6%)	108.0 (24.7%)	24.0 (21.2%)	18.0 (36.0%)	99.0 (23.2%)	51.0 (29.3%)
Intravenous infusion will not be effective for alleviating dry mouth in dying patients.	True: 175 (29.1%)	58.0 (28.6%)	117.0 (29.4%)	125.0 (28.5%)	29.0 (25.7%)	21.0 (42.0%) *	114.0 (26.7%)	61.0 (35.1%)
Total knowledge score, mean (SD)		7.67 (4.8)	8.08 (3.86)	7.78 (4.3)	8.44 (3.69)	8.2 (4.38)	7.89 (4.13)	8.07 (4.38)

Only 14 participants were considered knowledgeable (palliative care score above 90%) in questions about palliative medicine. According to the binary logistic regression, being a woman significantly reduced knowledge by 0.29 times. However, none of the participants were knowledgeable of palliative medicine being affected by any other predicted factor (Table 3).

**Table 3:** Determinants of knowledge about palliative medicine among the participants NA: not applicable

Variables		Not knowledgeable (N = 587)	Knowledgeable (N = 14)	OR (95% CI)	P value
Gender	Male	194.0 (33.0%)	9.0 (64.3%)	1	
	Female	393.0 (67.0%)	5.0 (35.7%)	0.29 (0.09-0.87)	0.027
Living site	City	416.0 (70.9%)	11.0 (78.6%)	1	
	Rural area	171.0 (29.1%)	3.0 (21.4%)	0.74 (0.2-2.76)	0.65
Specialty	Medical student	429.0 (73.1%)	9.0 (64.3%)	1	
	Resident	108.0 (18.4%)	5.0 (35.7%)	2.07 (0.67-6.4)	0.21
	Nurse or nursing student	50.0 (8.5%)	0.0 (0.0%)	NA	
Social status	Single	528.0 (89.9%)	11.0 (78.6%)	1	
	Married	56.0 (9.5%)	2.0 (14.3%)	8.08 (0.67-98.22)	0.1
	Divorced	3.0 (0.5%)	0.0 (0.0%)	NA	
	Widowed	0.0 (0.0%)	1.0 (7.1%)	NA	
Economic status	Bad	54.0 (9.2%)	2.0 (14.3%)	1	
	Average	296.0 (50.4%)	5.0 (35.7%)	0.3 (0.02-3.99)	0.36
	Good	206.0 (35.1%)	6.0 (42.9%)	0.59 (0.05-6.9)	0.68
	Excellent	31.0 (5.3%)	1.0 (7.1%)	NA	
Year of study (If you are a student)	First year	8.0 (1.9%)	2.0 (28.6%)	1	
	Second year	50.0 (11.8%)	2.0 (28.6%)	0.4 (0.03-5.47)	0.49
	Third year	77.0 (18.1%)	0.0 (0.0%)	NA	
	Fourth year	60.0 (14.1%)	2.0 (28.6%)	0.27 (0.02-3.71)	0.33
	Fifth year	133.0 (31.3%)	0.0 (0.0%)	NA	
	Sixth year	97.0 (22.8%)	1.0 (14.3%)	0.06 (0.003-1.4)	0.08

Table 4 results show that almost all participants (89.7%) agreed with the statement that "Pain relievers should be given as needed to terminally ill patients," followed by "Spiritual care must include counseling for the terminally ill patient" (84.3%) and "Patients have the right to determine their own degree of psychosocial intervention" (81%). However, about two-thirds of the participants (34.1%) disagreed that a patient's self-report of pain is a more accurate indicator of pain than a doctor's or nurse's rating of pain.

**Table 4:** Description of the attitude of the participants regarding palliative medicine

Questions	Strongly agree	Agree	Neutral	Disagree	Strongly agree
Pain at the end of life is an inevitable part of the dying process.	103 (17.1%)	189 (31.4%)	159 (26.5%)	129 (21.5%)	21 (3.5%)
Pain relievers should be given as needed to terminally ill patients.	244 (40.6%)	295 (49.1%)	40 (6.7%)	22 (3.7%)	0
Spiritual care must include counseling for the terminally ill patient.	279 (46.4%)	228 (37.9%)	69 (11.5%)	22 (3.7%)	3 (0.5%)
I don't like talking about death with patients.	203 (33.8%)	192 (31.9%)	155 (25.8%)	41 (6.8%)	10 (1.7%)
Palliative care should be the standard medical treatment for terminally ill patients.	186 (30.9%)	251 (41.8%)	128 (21.3%)	32 (5.3%)	4 (0.7%)
Patients should have the right to determine their own degree of medical intervention.	179 (29.8%)	269 (44.8%)	100 (16.6%)	50 (8.3%)	3 (0.5%)
Addiction to oral morphine is not a serious problem given that terminally ill patients have a short time to live.	107 (17.8%)	186 (30.9%)	180 (30.0%)	110 (18.3%)	18 (3.0%)
Discussions on end-of-life care should be postponed until no other effective treatment is available.	101 (16.8%)	198 (32.9%)	177 (29.5%)	97 (16.1%)	28 (4.7%)
A pain rating by a doctor or nurse is a more valid measure of pain than a patient's self-report.	89 (14.8%)	130 (21.6%)	177 (29.5%)	166 (27.6%)	39 (6.5%)
Complete pain relief is a reasonable goal even when the pain is not caused by a condition such as cancer.	162 (27.0%)	246 (40.9%)	130 (21.6%)	56 (9.3%)	7 (1.2%)
Patients have the right to determine their own degree of psychosocial intervention.	198 (32.9%)	289 (48.1%)	84 (14.0%)	25 (4.2%)	5 (0.8%)
The most appropriate person to take end-of-life decisions and measures is the patient's primary caregiver (the first person to deal with the patient).	103 (17.1%)	176 (29.3%)	195 (32.4%)	114 (19.0%)	13 (2.2%)
The patient should feel uncomfortable before receiving the second dose of pain relievers.	69 (11.5%)	128 (21.3%)	200 (33.3%)	161 (26.8%)	43 (7.2%)
Patients should be kept in a pain-free condition.	183 (30.4%)	245 (40.8%)	132 (22.0%)	38 (6.3%)	3 (0.5%)
As a general rule, it is preferable not to talk about death with the terminally ill.	220 (36.6%)	178 (29.6%)	103 (17.1%)	72 (12.0%)	28 (4.7%)

In addition, there was a significant difference in attitude scores between city and country residents (P = 0.004). Moreover, while there was no statistically significant difference between people of different economic statuses, the score was statistically different between specialties, social status, and years of study. Regarding the logistic regression, there were no other predictors for the participants' attitudes other than their year of study, with fifth-year students being 8.06 times more likely to have a positive attitude than first-year students (Table 5).

**Table 5:** Determinants of attitude regarding palliative medicine among the participants NA: not applicable

Variables	Attitude categories			Attitude score mean (SD)	OR (95% CI)	P value
	Positive (≥75% of score) (N=12)	Neutral (70%˃, ≥50%) (N=287)	Negative (˂50%) (N=302)			
Gender				P value= 0.7		
Male	4.0 (33.3%)	101.0 (35.2%)	98.0 (32.5%)	23 (5.22)	1	
Female	8.0 (66.7%)	186.0 (64.8%)	204.0 (67.5%)	22.9 (4.92)	1.14 (0.32-4.03)	0.84
Living site				P value= 0.004		
City	10.0 (83.3%)	216.0 (75.3%)	201.0 (66.6%)	23.3 (5.13)	1	
Rural area	2.0 (16.7%)	71.0 (24.7%)	101.0 (33.4%)	22 (4.62)	3.08 (0.65-14.67)	0.16
Specialty				P value˂ 0.001		
Medical student	10.0 (83.3%)	221.0 (77.0%)	207.0 (68.5%)	23.2 (4.95)	1	
Resident	0.0 (0.0%)	55.0 (19.2%)	58.0 (19.2%)	22.8 (4.76)	NA	
Nurse or nursing student	2.0 (16.7%)	11.0 (3.8%)	37.0 (12.3%)	20.5 (5.59)	0.67 (0.12-3.86)	0.65
Social status				P value˂ 0.001		
Single	11.0 (91.7%)	272.0 (94.8%)	256.0 (84.8%)	23.2 (4.9)	1	
Married	1.0 (8.3%)	15.0 (5.2%)	42.0 (13.9%)	20.9 (5.48)	0.91 (0.09-9.55)	0.94
Divorced	0.0 (0.0%)	0.0 (0.0%)	3.0 (1.0%)	16 (1)	NA	
Widowed	0.0 (0.0%)	0.0 (0.0%)	1.0 (0.3%)	15 (NA)	NA	
Economic status				P value= 0.29		
Bad	1.0 (8.3%)	26.0 (9.1%)	29.0 (9.6%)	22.8 (5.41)	1	
Average	10.0 (83.3%)	132.0 (46.0%)	159.0 (52.6%)	22.7 (5.37)	0.49 (0.06-4.12)	0.51
Good	1.0 (8.3%)	111.0 (38.7%)	100.0 (33.1%)	23.1 (4.42))	3.87 (0.22-67.06)	0.35
Excellent	0.0 (0.0%)	18.0 (6.3%)	14.0 (4.6%)	23.4 (4.74)	NA	
Year of study (If you are a student)				P value= 0.002		
First year	2.0 (16.7%)	3.0 (1.4%)	5.0 (2.5%)	24.6 (9.88)	1	
Second year	3.0 (25.0%)	26.0 (11.7%)	23.0 (11.6%)	24.4 (6.14)	4.08 (0.59-28.4)	0.16
Third year	0.0 (0.0%)	46.0 (20.7%)	31.0 (15.7%)	23.4 (4.13)	NA	
Fourth year	3.0 (25.0%)	35.0 (15.8%)	24.0 (12.1%)	24.2 (5.98)	4.92 (0.71-34.1)	0.11
Fifth year	4.0 (33.3%)	66.0 (29.7%)	63.0 (31.8%)	23.3 (4.5)	8.06 (1.28-50.8)	0.026
Sixth year	0.0 (0.0%)	46.0 (20.7%)	52.0 (26.3%)	22.3 (4.34)	NA	

## Discussion

Palliative care is active, continuing care provided by an interdisciplinary team at an institution or at home that is primarily focused on the patient's comfort, generally at the terminal stage of life [20]. Palliative care aims to prevent and relieve physical pain, unpleasant symptoms, and psychological distress. Palliative care is available to all patients suffering from serious illnesses, regardless of prognosis, age, disease stage, treatment choice, or specialty. Its use has recently been widely incorporated in the multidisciplinary management of patients with cancer and neuropathic pain [21]. The goal of this study was to assess Syrian healthcare workers' knowledge of palliative care to improve the health of patients facing life-threatening conditions.

According to the findings, palliative care knowledge was found to be poor and clearly insufficient among the population, with only 14 participants (2.3%) being knowledgeable. There were no significant differences in reported living cities or specialties. Furthermore, getting enough sleep was the most correctly answered question for pain management purposes in the philosophy section, with 84.2% of the respondents answering correctly. The correct response rate for further addiction caused by long-term palliative care was 5.3%. These findings are consistent with those reported by Kumar et al. in an Indian study assessing palliative care knowledge among Indian nurses [22] and a Saudi Arabian study conducted by Aboshaiqah and Ahmad among Saudi nurses [23]. Nearly 90% of the participants agreed that painkillers should be administered to terminally ill patients as needed for palliative care indications. These findings closely correlate with those of a Chinese study by Cheng et al., who found that oncology patients strongly supported the use of palliative medications in end-of-life care [24].

The patients’ right to determine their own level of psychosocial intervention and need for palliative care was strongly supported by nearly three-quarters of the participants (74.6%). However, 34.1% of the participants agreed that a doctor's or nurse's pain rating was a more valid measure of pain than a patient's self-report, confirming the critical importance of self-pain assessment in pain management and palliative care use. In terms of attitudes toward palliative care, fifth-year medical students significantly outperformed first-year students by 8.06 times. These findings were also found in a study conducted in the United Arab Emirates by Ibrahim Halah and colleagues, who discovered that newly graduated students had a lower attitude toward and knowledge of palliative care when compared to experimented physicians [25]. As a result, first-year medical students must be involved in palliative care education to improve it and learn how to use it in clinics.

The findings clearly indicate that Syrian healthcare workers' knowledge of palliative care was poor and insufficient. This lack of knowledge must be corrected and rapidly improved, not only because it is one of the Syrian medical education improvement aspects but also because the incorporation of palliative care in multidisciplinary care management is now widely used in multiple fields and for several diseases, such as cancer, chronic serious illness, kidney and heart failure, and rheumatoid arthritis. As a result, patients will receive better care, and even if life expectancy does not increase, their quality of life will undoubtedly improve [26].

Palliative care knowledge can be improved through simple, logical, and correct decisions. First, its inclusion in the early medical and nursing education curriculum and undergraduate studies ensures mastery following graduation [27]. Second, comprehensive clinical education, seminars, webinars, conferences, and palliative care research projects that build on the existing research literature are important factors in its emergence as a critical topic among healthcare professionals [28,29]. Furthermore, improving doctor-patient communication is a significant step toward understanding patients' needs and working to ensure their satisfaction, particularly among end-of-life patients [30]. Finally, several healthcare practitioners have assured the availability of an approach to de-specialize palliative care from a specific field to multiple specialties, which will broaden its knowledge and strengthen its practice [31].

Strengths and limitations

The availability of an inspector who oversaw the data collection procedure to eliminate multiple auto and random replies was one of the primary strengths of this study. In addition, to reduce bias, the poll was disseminated across all Syrian regions. This study has a few limitations. The use of a cross-sectional online poll did not demonstrate any causality. Other issues arise when people do not have an Internet connection or a device will not be able to fill out an online survey. Furthermore, no study in the Arab world has verified our questionnaire.

## Conclusions

The knowledge of palliative care among Syrian healthcare practitioners is poor. This knowledge gap must be addressed quickly, not only because it is one of the concerns that Syrian medical schools must address but also because the use of palliative care in multidisciplinary care coordination is now common across many medical disorders. With 84.2% of responders properly replying, getting enough sleep was the most accurately answered question in the philosophy section portion for pain treatment aims. The correct response rate for long-term palliative care medication use leading to addiction was 5.3%. This study underlines the need for education and awareness-raising initiatives, particularly in countries with low or middle incomes.
